# 594 23 Years Later: Long-term Functional Outcomes of 9/11 Survivors

**DOI:** 10.1093/jbcr/iraf019.223

**Published:** 2025-04-01

**Authors:** Anna Vaeth, Makayla Kochheiser, Lucy Wei, Nancy Qin, Grant Black, Nicholas Vernice, David Janhofer, Philip Chang, Palmer Bessey, David Otterburn

**Affiliations:** Weill Cornell Medicine; Weill Cornell Medicine; Weill Cornell Medicine; Weill Cornell Medicine; Weill Cornell Medicine; Weill Cornell Medicine; Weill Cornell Medicine; Weill Cornell Medicine; Weill Cornell Medicine; Weill Cornell Medicine

## Abstract

**Introduction:**

The September 11th attacks were a unique disaster with numerous patients and extensive injury burden. The aim of this study was to provide an update on the long-term health and functional recovery of victims treated at a burn center following the September 11th attacks.

**Methods:**

A mixed methods approach was completed by using a cross-sectional analysis with a survey to study health outcomes and a qualitative interview for each patient. All patients were treated at our institution’s burn center for burn injuries sustained during the September 11th attacks. Interviews were reviewed for trends in burn injury recovery and functionality.

**Results:**

There were 14 patients treated and discharged from the burn center following the September 11th attacks. Of those patients, four patients participated in the study including three males and one female. The average age was 63 years (range:57-73) and average total body surface area burned was 33.1% (range:3%-80%). One patient was burned in the elevator in the North Tower and one patient was burned in the lobby of North Tower during the impact of American Airlines Flight 11. Two patients were burned outside by debris. Following initial recovery of their burn injuries with wound care, two patients required operative contracture release and one patient required surgery to address heterotopic ossification. Additionally, one patient required extensive orthopedic surgeries to improve hand function. All four patients returned to work after their injuries with modifications. One patient took summers off for 3 years post-attacks due to heat strokes, one patient worked fewer hours, and two patients switched careers. One patient switched from iron working to tunnel building due to fear of dropping tools from heights. One patient switched from an executive position to pursuing additional degrees and running a non-profit organization. Of the four patients, two were currently retired. All patients returned to hobbies they previously enjoyed such as golfing, walking, basketball, and exercising.

**Conclusions:**

Our study showed that burn victims from the September 11th attacks were able to return to work and to enjoy activities they previously enjoyed following their injuries. Adverse outcomes of their burns that required further surgery included contractures, heterotopic ossification, and hand functionality. Overall, our study showed positive trends in long-term recovery following a mass casualty event.

**Applicability of Research to Practice:**

This study seeks to provide new information on how the intersection of personal injury and large-scale disaster affects long-term physical recovery. The study allows us to gain a deeper understanding of each patient’s experience in the long-term recovery phases. Although the September 11th attacks were a unique disaster, this study helps us predict how patients may recover overtime from similar large-scale disasters in the future.

**Funding for the Study:**

N/A

